# Supplementary material for: The Selective α1 Antagonist Tamsulosin Alters ECM Distributions and Cellular Metabolic Functions of ARPE 19 Cells in a Concentration-Dependent Manner
Source: Bioengineering (Basel). 2022 Oct 14;9(10):556. doi: 10.3390/bioengineering9100556 (PMC9598783; doi:10.3390/bioengineering9100556)
Supplement: Supplementary file 1 [file bioengineering-09-00556-s001.zip › bioengineering-1911117-supplementaty.pdf]

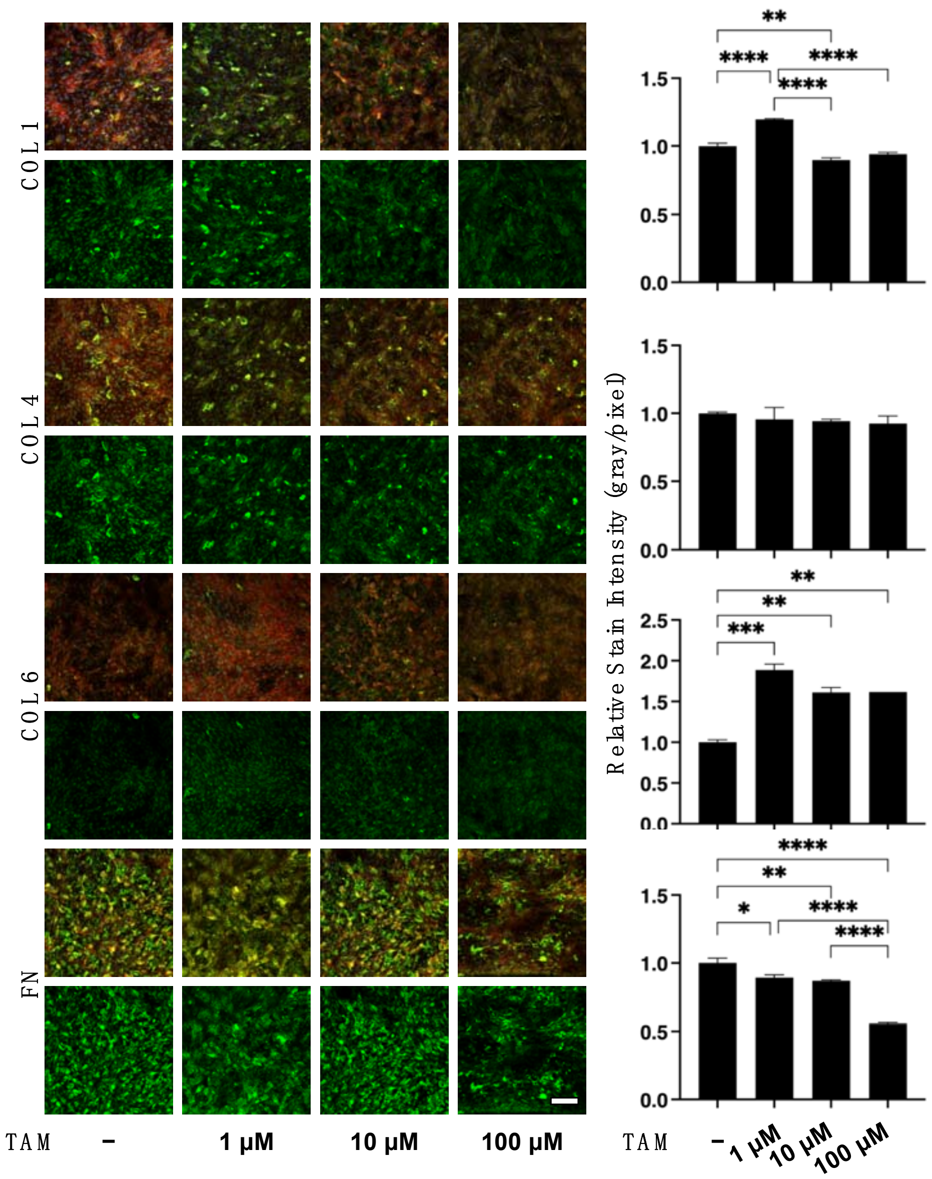

Figure S1: Representative confocal images showing the expression of ECMs in 2D ARPE19 monolayers under several conditions.

2D ARPE 19 monolayers at Day 5 in the absence or presence of 1 mM, 10 mM or 100 mM tamsulosin (TAM) were subjected to immunohistochemistry analyses. Representative immunolabeling images by specific antibodies against collagen 1 (COL 1), collagen 4 (COL 4), collagen 6 (COL6), or fibronectin (FN) (green), DAPI (blue) and Phalloidin (red) are shown in panel A (scale bar: 100 μm). The staining intensities of the labeling of each ECM protein were plotted in panel B. All experiments were performed in duplicate using fresh preparations consisting of 5 spheroids each. Data are presented as the arithmetic mean ± standard error of the mean (SEM). * P<0.05, ** P<0.01, *** P<0.005, **** P<0.001 (ANOVA followed by a Tukey's multiple comparison test).

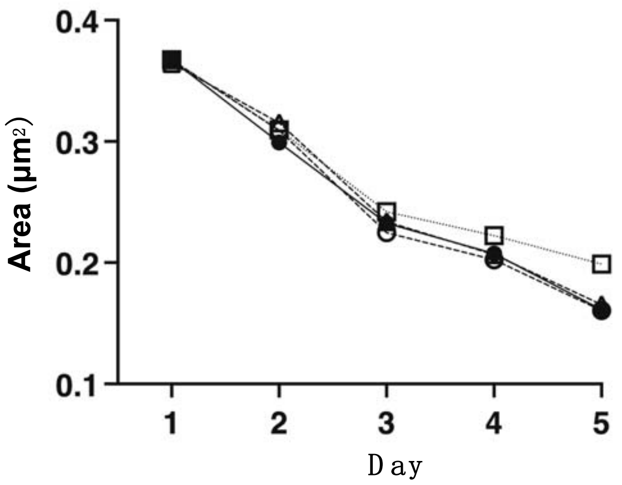

Figure S2: Time course of changes in the mean sizes of the 3D ARPE 19 spheroids during 5 days culture.

The fluctuation in the mean sizes of the 3D ARPE19 spheroids of non-treated controls (CONT), or in the presence of 1 mM, 10 mM or 100 mM tamsulosin (TAM) during 5 days spheroid culture are plotted.

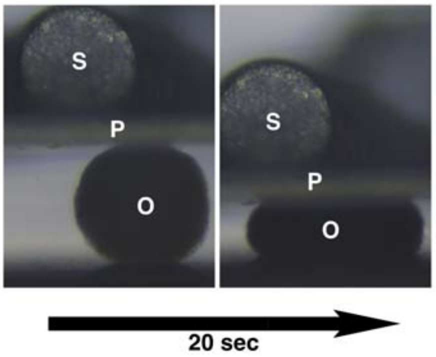

Figure S3: The measurement of the physical stiffness using a microsqueezer.

A single living 3D ARPE19 spheroid at Day 5 was compressed and the force (μN) required to induce a 50 % deformity of every single spheroid out of 15-20 freshly prepared 3D spheroids were measured over a period of 20 seconds (panel A, O; 3D spheroid, P; compressing plate, S; pressure sensor).

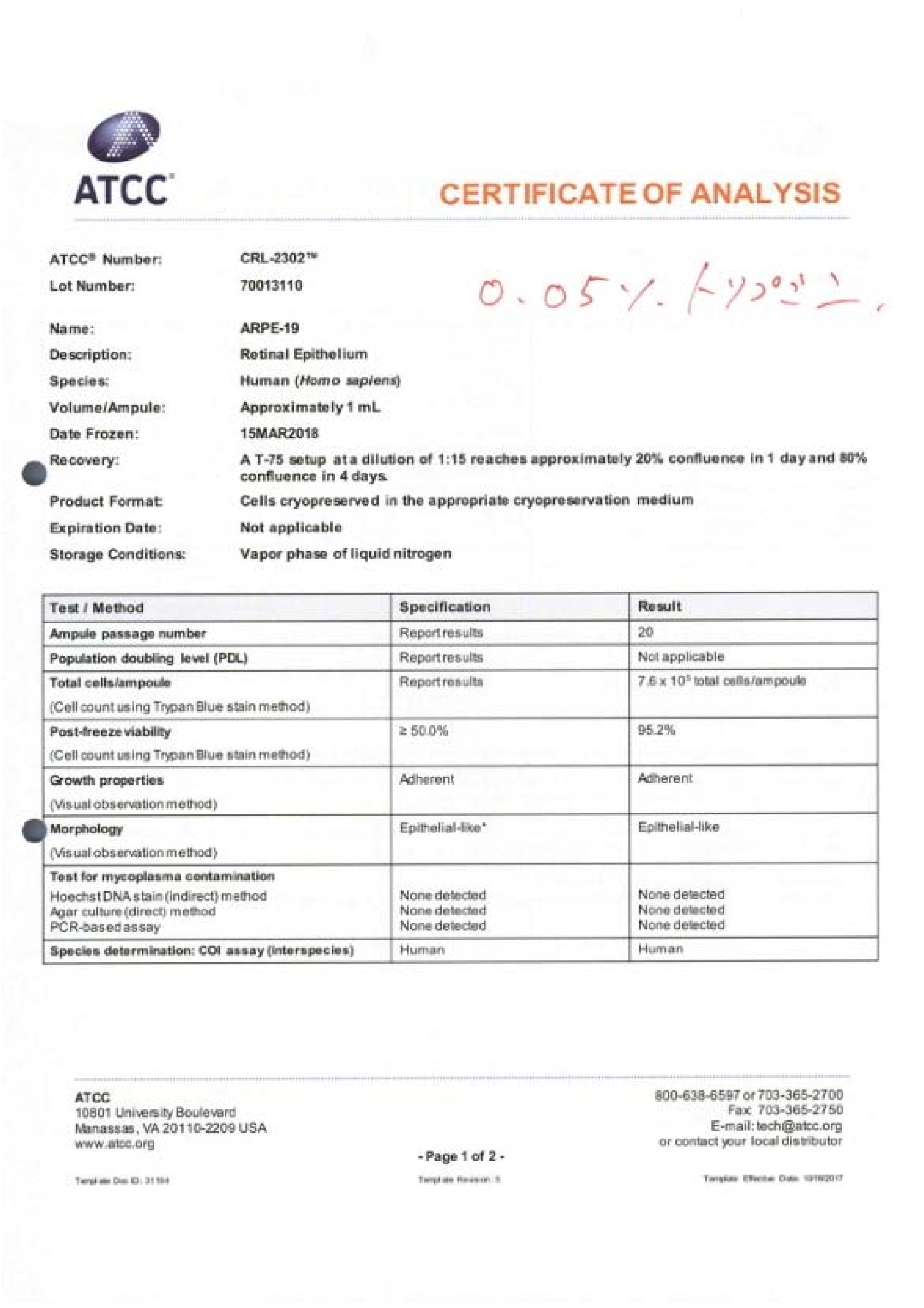

ATCC

CERTIFICATE OF ANALYSIS

ATCC® Number: CRL-2302™

Lot Number: 70013110

0.05%. トリプシン.

Name: ARPE-19

Description: Retinal Epithelium

Species: Human (*Homo sapiens*)

Volume/Ampule: Approximately 1 mL

Date Frozen: 15MAR2018

Recovery: A T-75 setup at a dilution of 1:15 reaches approximately 20% confluence in 1 day and 80% confluence in 4 days.

Product Format: Cells cryopreserved in the appropriate cryopreservation medium

Expiration Date: Not applicable

Storage Conditions: Vapor phase of liquid nitrogen

Test / Method	Specification	Result
**Ampule passage number**	Report results	20
**Population doubling level (PDL)**	Report results	Not applicable
**Total cells/ampoule**<br>(Cell count using Trypan Blue stain method)	Report results	7.6×10^{5} total cells/ampoule
**Post-freeze viability**<br>(Cell count using Trypan Blue stain method)	≥ 50.0%	95.2%
**Growth properties**<br>(Visual observation method)	Adherent	Adherent
**Morphology**<br>(Visual observation method)	Epithelial-like*	Epithelial-like
**Test for mycoplasma contamination**<br>Hoechst DNA stain (indirect) method<br>Agar culture (direct) method<br>PCR-based assay	 None detected<br>None detected<br>None detected	 None detected<br>None detected<br>None detected
**Species determination: COI assay (interspecies)**	Human	Human

ATCC
10801 University Boulevard
Manassas, VA 20110-2209 USA
www.atcc.org

800-638-6597 or 703-365-2700
Fax: 703-365-2750
E-mail: tech@atcc.org
or contact your local distributor

- Page 1 of 2 -

Table S1: The Quantitative PCR primers
